# Impact of the Great East Japan Earthquake on the Employment Status and Mental Health Conditions of Affected Coastal Communities

**DOI:** 10.3390/ijerph17218130

**Published:** 2020-11-03

**Authors:** Mitsuaki Katayanagi, Moe Seto, Naoki Nakaya, Tomohiro Nakamura, Naho Tsuchiya, Akira Narita, Mana Kogure, Yumi Sugawara, Akira Kodaka, Yusuke Utsumi, Hitomi Usukura, Yasuto Kunii, Atsushi Hozawa, Ichiro Tsuji, Hiroaki Tomita

**Affiliations:** 1 Department of Disaster Psychiatry, Graduate School of Medicine, Tohoku University, 2-1 Seiryo-Machi, Aoba-ku, Sendai 980-8573, Japan; mitsuaki.katayanagi.t8@dc.tohoku.ac.jp (M.K.); moe.seto518@gmail.com (M.S.); ysutsumi@med.tohoku.ac.jp (Y.U.); 2 Miyagi Disaster Mental Health Care Center, 2-8-21 Honcho, Aoba-ku, Sendai 980-0014, Japan; kodaka.akirasan@gmail.com; 3 Department of Psychiatry, Tohoku University Hospital, 1-1 Seiryo-Machi, Aoba-ku, Sendai 980-8574, Japan; 4 Department of Psychiatry, Graduate School of Medicine, Tohoku University, 2-1 Seiryo-Machi, Aoba-ku, Sendai 980-8575, Japan; 5 Department of Preventive Medicine and Epidemiology, Tohoku Medical Megabank Organization, Tohoku University, 2-1 Seiryo-Machi, Aoba-ku, Sendai 980-8573, Japan; nakaya-thk@umin.ac.jp (N.N.); tnakamura@megabank.tohoku.ac.jp (T.N.); nahot@megabank.tohoku.ac.jp (N.T.); narita-a@megabank.tohoku.ac.jp (A.N.); m-kogure@med.tohoku.ac.jp (M.K.); hozawa-thk@umin.ac.jp (A.H.); 6 Department of Health Sciences, Saitama Prefectural University, 820 Sannomiya, Koshigaya-City, Saitama 343-8540, Japan; 7 Department of Public Health, Graduate School of Medicine, Tohoku University, 1-1 Seiryo-machi, Aoba-ku, Sendai 980-8574, Japan; yumi1717@med.tohoku.ac.jp (Y.S.); tsuji1@med.tohoku.ac.jp (I.T.); 8 Department of Disaster Psychiatry, International Research Institute of Disaster Science, Tohoku University, 2-1 Seiryo-Machi, Aoba-ku, Sendai 980-8573, Japan; usukura@med.tohoku.ac.jp (H.U.); yasuto.kunii@gmail.com (Y.K.)

**Keywords:** cross-sectional study, disaster, employment status, mental health, structural industry characteristics

## Abstract

The Great East Japan Earthquake devasted the old community in coastal areas characterized by primary industry. The number of unemployed people increased from 150,000 to 190,000 after the earthquake. All of the adult residents of Shichigahama (18 years old or older), located in the coastal area of the Miyagi prefecture, whose houses were totally or majorly damaged, were recruited for a survey conducted in October 2011. All of the residents who responded with written informed consent were included in this study. Among 904 individuals who had a job before the Great East Japan Earthquake, 19% became unemployed. Concerning gender and age, 9% of young men, 34% of elderly men, 21% of young women, and 49% of elderly women became unemployed. Concerning the type of industry, 38%, 15%, and 16% of people who had belonged to the primary, secondary, and tertiary industries, respectively, before the disaster became unemployed. Those who became unemployed exhibited a significantly higher risk of insomnia compared to those who maintained jobs. The study pointed out the severe impact of the Great East Japan Earthquake on populations who had belonged to the primary industry, especially among elderly women, and its effect on sleep conditions.

## 1. Introduction

The Great East Japan Earthquake that occurred in 2011 caused massive damage to the coastal area of northeastern Japan, killing more than 15,000 people. The disaster impacted the social environment, including the employment status and economic conditions of communities [1]. According to public data, the number of unemployed people increased from 150,000 to 190,000 after the earthquake [2].

It has been reported that disasters greatly affect employment status and economic conditions [3] and that employment status and economic conditions affected by disasters impact the mental health of the affected people [4,5]. Sleep status is an important factor closely related to mental health; sleep disturbances worsen mental health conditions, and vice versa [6,7,8]. Previous studies have also indicated that disasters affect the sleep status of the impacted people [9,10,11]. The effect of disasters on sleep status can be directly or indirectly (e.g., through altered mental health conditions) related to employment status, and changes in employment status after disasters can affect the sleep states of affected people.

The relationships among disasters, employment status, economic conditions, and mental health conditions have been evaluated in communities affected by disasters. A study one year after the 2001 terrorist attacks on the United States suggested that unemployment and exposure to adverse work conditions, particularly high levels of perceived work stress, could be important determinants of the persistence of posttraumatic stress after a disaster [12]. Another study after the explosion of the fertilizer plant in Toulouse, France, in 2001 documented that the impact on the workplace and socioeconomic conditions were associated with the symptoms of posttraumatic stress disorder [13]. A mixed-method study of low-income mothers who survived Hurricane Katrina in 2005 documented that improved employment opportunities were found to underlie resilience and other positive mental health outcomes [14].

While there might be common relationships among employment status, economic conditions, and mental health conditions in communities affected by a disaster regardless of its type, there might also be relationships among employment status, economic conditions, and mental health conditions unique to the type of disaster. It is necessary to evaluate the relationships among employment status, economic conditions, and mental health conditions in each disaster. The impact of the Great East Japan Earthquake on employment status and the relationships among employment status, mental health conditions, and sleep status in communities affected by the Great East Japan Earthquake have not been fully evaluated. It is important to collect this information, including data about the mental health conditions of residents, to proceed with the recovery of the affected communities. The impact of disaster and recovery processes can be influenced by structural industry characteristics (the proportion of people who work in the primary, secondary, and tertiary industries) of the affected community. As a specific issue concerning coastal areas severely damaged by the Great East Japan Earthquake, the primary industries of these communities were fisheries and agriculture, and many people who were older than 65 years old were engaged in these industries [15], therefore, the disaster might have especially impacted the employment status and mental health conditions of the primary industry workers who were older than 65.

The aims of this study were to profile the impact of the Great East Japan Earthquake on employment status and to investigate the associations between employment status and mental health conditions considering the structural industry characteristics of the affected coastal area. The total subjects were classified into three groups: (1) people who had not worked before the earthquake; (2) people who became unemployed after the earthquake; and (3) people who continued to work after the earthquake; differences in sleep conditions, psychological distress, and posttraumatic stress reactions among these three groups were evaluated to test the hypothesis that mental health conditions of people who became unemployed after the earthquake might be worse than those of people who had not worked before the earthquake or people who continued to work after the earthquake.

## 2. Materials and Methods

### 2.1. Subjects

An explanation of the survey, an informed consent form, and questionnaires were distributed to all of the adult residents of Shichigahama (18 years old or older), located in the coastal area of the Miyagi prefecture at the onset of the Great East Japan Earthquake, whose houses were totally destroyed or severely damaged by the disaster based on the publicly standardized assessment by the local government for damage certificates. The survey was conducted in October 2011 as part of an annual survey conducted as a collaborative health survey between Shichigahama and Tohoku University. All of the residents who responded with written informed consent were included in this study.

The survey consisted of self-administered questionnaires to grasp the health and life conditions of the affected residents. Of the 2456 residents who satisfied the above criteria, 1550 (63.1%) completed the survey with written informed consent. Among them, 1494 subjects (710 men and 784 women) completed the questionnaire about employment status before the earthquake, which was subjected to analysis.

### 2.2. Consideration of Aged Populations

The average age of the subjects was 55.3 years old (SD = 18.2). Considering the characteristics of the coastal communities inundated by the tsunami, the employment status of people aged 65 years old and older was considered because a considerable number of residents worked in the fishery or agriculture industry, and this population tended to work even after turning 65 years old, while the majority of employed persons retire at 65 years old at the latest in Japan. Of the total residents, the number of elderly people aged 65 years old or older was 841. Among them, 566 (67.3%) responded to the survey with written informed consent. Among them, 526 (229 men and 297 women) completed the questionnaire for employment status before the disaster.

### 2.3. Employment Status

Changes in the employment status of the subjects before and after the earthquake were profiled for each subpopulation classified by age, gender, and job type. The subjects were asked to select “yes” or “no” for a question about employment status: “did you have a job before the earthquake”? If they answered “yes”, they were asked to select one or more from the occupational categories “agriculture”, “fishery”, “mining”, “construction”, “manufacturing”, “electricity, gas, water supply”, “information or communication”, “transportation or postal”, “wholesale or retail”, “financial or insurance”, “service industries (restaurants, tourism industry, accommodation)”, “education, medical, welfare, or public affairs”, and “other.” The subjects were then asked to select either “altered” or “no change” regarding changes in employment status caused by the earthquake: “was your employment status changed by the earthquake?” If they selected “altered”, they were asked to select one or more from the following options: “started a new job (including job change)”, “unemployed”, “stayed employed with increased earnings”, “stayed employed with decreased earnings”, and “other” for the question “how did it change”? People who answered that their employment status was altered by the earthquake and they became unemployed were regarded as having unemployed status due to the earthquake. Among the occupations, “agriculture” and “fishery” were classified as the primary industries; the “mining”, “construction”, and “manufacturing” industries were classified as the secondary industries; and the “electricity, gas, water supply”, “information or communication”, “transportation or postal”, “wholesale or retail”, “financial or insurance”, “service industries (restaurant, sightseeing industry, accommodation)” industries, and “education, medical care, welfare, or public affairs” industries were classified as tertiary industries, according to the Japan Standard Industrial Classification [16].

### 2.4. Mental Health Conditions

This survey’s questionnaire also included inquiries about perceived economic conditions; habits of eating, smoking, and drinking alcohol; physical conditions, sleep conditions evaluated by the Athens insomnia scale (AIS) [17,18]; psychological distress evaluated by the Kessler 6 (K-6) scale [19,20]; posttraumatic stress reactions evaluated by the revised impact-of-event scale (IES-R) [21,22].

The AIS is a self-reported questionnaire to evaluate sleep difficulty and is scored with a four-point scale. The first five items assess sleep induction, awakenings during the night, final awakening, total sleep duration, and sleep quality. The next three items assess well-being, functional capacity, and sleepiness during the day [17]. The Japanese-language version of the AIS has been validated [18].

The K-6 has been widely used for epidemiological surveys detecting problems in mood and anxiety. The participants were asked whether during the past 30 days they had felt “nervous”, “hopeless”, “restless or fidgety”, “so depressed that nothing could cheer you up”, “that everything was an effort”, or “worthless.” Each question was rated on a five-point Likert scale from zero (none of the time) to four (all of the time), with higher scores signifying worse mental health conditions [19]. The Japanese version of the K-6 has been validated as an effective method for identifying psychological distress [20].

The IES-R is a 22-item self-rating scale to evaluate traumatic stress symptoms developed by Weiss [21]. The scale consists of three subscales: intrusion, avoidance, and hyperarousal. The Japanese-language version of the IES-R has been validated [22].

### 2.5. Statistical Analyses

The total subjects were classified into three groups: (1) people who had not worked before the earthquake; (2) people who became unemployed after the earthquake; and (3) people who continued to work after the earthquake; differences in the AIS, K-6, and IES-R among these three groups were evaluated by one-way analysis of variance using JMP statistical analysis software (SAS Institute Inc, Cary, NC, USA) [23] to test the hypothesis that mental health conditions of people who became unemployed after the earthquake might be worse than those of people who had not worked before the earthquake or people who continued to work after the earthquake.

The study was conducted following the protocol approved by the Tohoku University Ethics Committee (approval number 2018-1-535).

## 3. Results

Among the total subjects who responded to employment status before the Great East Japan Earthquake (*n* = 1494), 904 (60.5%) worked before the earthquake. Regarding the proportion of people who worked before the earthquake by gender and age, 84.4% of young men and 67.4% of young women (both younger than 65 years old) worked, while 42.8% of elderly men and 24.2% of elderly women (both 65 years old or older) worked before the earthquake (Table 1).

Among subjects who had worked before the earthquake, 173 (19.1%) became unemployed after the earthquake. The proportion of people who became unemployed after the earthquake by gender and age was 9.1% young men, 20.7% young women, 33.7% elderly men, and 48.6% elderly women. The impact of the earthquake on employment status prevailed in each group, particularly in the female and elderly subpopulations (Table 2).

The proportions of subjects who engaged in primary industries, such as agriculture and fishery, before the disaster were 11.5% young men, 9.3% young women, 57.1% elderly men, and 49.3% elderly women (Table 3). The proportion of people who became unemployed after the disaster in each type of industry was 37.9% for the primary industry, 15.2% for the secondary industry, and 16.1% for the tertiary industry. There were significant differences in the distributions of people who continued to work at the same workplace after the earthquake in each industry between young people and elderly people (*p* < 0.01). The proportions of people who became unemployed after the disaster among elderly men (39.1%) and young women (51.5%) were prominent in the primary industry. In the secondary industry, the number of elderly men who became unemployed after the disaster (43.8%) was prominent. In the tertiary industry, the unemployment proportion among elderly women (55.2%) was prominent (Table 4).

Regarding perceived alterations in income for the 683 subjects who continued to work in the same workplace after the earthquake, 37 (5.4%) reported increased income, while 185 (27.1%) reported decreased income. Nearly one-fifth (18.4%) of young men who remained engaged in the secondary industry reported increased income after the earthquake, while the proportions were only 6.9% of young men who remained engaged in the primary industry and 5.4% of young men who remained engaged in the tertiary industry. In contrast, 62.1% of young men who remained engaged in the primary industry, 14.9% of young men who remained engaged in the secondary industry, and 25.5% of young men who remained engaged in the tertiary industry reported decreased income. Similarly, 43.8% of elderly men, 70.0% of young women, and 50.0% of elderly women who were engaged in the primary industry reported decreased income after the earthquake (Table 5).

The average AIS score, an indicator of insomnia, for the group that had not worked before the earthquake was 4.76; for the group that became unemployed after the earthquake, it was 6.11; and for the group that continued to work after the earthquake, it was 4.57 (SD = 4.05, 3.99, and 3.50, respectively). There was a significant difference among the three groups (*p* < 0.01). Tukey–Kramer’s HSD tests showed a significant difference between those who became unemployed after the earthquake and those who continued to work since the earthquake (*p* < 0.01). A significant difference was also shown between the group who had not worked before the earthquake and the group who became unemployed after the earthquake (*p* < 0.01). As there was a significant difference in the age of subjects that made up each group (*p* = 0.02), analysis of covariance was applied to the data using age as a confounding factor. The analyses indicated that there was no significant interaction between age and employment status (*p* = 0.37), while the difference in AIS among the groups remained statistically significant (*p* < 0.01).

The relationship between employment status and mental health conditions in the 2011 survey data was evaluated using a one-way analysis of variance. The average score of K-6, an indicator of psychological distress, for the group that had not worked before the earthquake was 5.26; for the group that became unemployed after the earthquake, it was 5.90; and for the group that continued to work after the earthquake, it was 4.97 (SD = 4.84, 4.86, and 4.56, respectively). There was no significant difference among the three groups (*p* = 0.08).

The average score of the IES-R, an indicator of posttraumatic stress reactions, for the group who had not worked before the earthquake was 21.07; for the group who became unemployed after the earthquake, it was 22.22; and for the group who continued to work after the earthquake, it was 17.94 (SD = 16.61, 15.57, and 14.77, respectively). There was a significant difference among the three groups (*p* < 0.01). Tukey–Kramer’s HSD tests showed a significant difference between those who were unemployed after the earthquake and those who continued to work (*p* < 0.01). A significant difference was also indicated between the group that had not worked before the earthquake and the group that continued to work after the earthquake (*p* = 0.01). As there was a significant difference in the age of subjects of each group (*p* < 0.01), analysis of covariance was applied to the data using age as a confounding factor, showing that there was no significant difference in the IES-R among the three groups (*p* = 0.22; Table 6).

## 4. Discussion

First, this study aimed to profile the impact of the Great East Japan Earthquake on employment status. The proportion of people who became unemployed after the earthquake was 19.1% among the community in general. Concerning gender and age proportions, 9.1% of young men and 20.7% of young women became unemployed, while much larger proportions were found for the elderly (33.7% of elderly men and 48.6% of elderly women). For the proportions of becoming unemployed after the earthquake for each type of industry regardless of age, 15.2% in the secondary industry group and 16.1% in the tertiary industry group became unemployed, while a much larger proportion (37.9%) in the primary industry became unemployed. The data highlighted the severe impact of the disaster on affected residents who worked in the primary industry, leading to a large unemployed proportion of elderly populations. The data also indicated that the unemployment proportion of women was much larger than that of men, especially among elderly populations.

Among those who continued to work in the same workplace, nearly half of the primary industry workers reported decreased income after the disaster, showing a greater impact of the disaster on those who worked in the primary industry compared with those who worked in the secondary and tertiary industries. It was also remarkable that the majority of elderly people reported declined income regardless of the type of industry. Considered together, the data highlighted the severe impact of the disaster on both the employment and economic statuses of elderly people, reflecting that a considerable proportion of elderly people was engaged in self-employed fisheries and agriculture with no retirement system before the Great East Japan Earthquake in typical communities affected by the disaster.

According to a government report in 2012, the percentages of employed elderly men and women (65 years old or older) in Japan were 27.8% and 13.1%, respectively [24]. Moreover, the town of Shichigahama is renowned for its fishing industry, including aquacultures of seaweed, sea urchins, and abalone, and there are many rice fields in the town. Many elderly people belonged to these industries. The survey indicated that the proportions of elderly men and women who were employed in Shichigahama before the Great East Japan Earthquake were 42.8% and 24.2%, respectively, which were much higher than for the average population in Japan. Before the earthquake, larger proportions of the young population (younger than 65 years old, both male and female) in Shichigahama were employed in the secondary and tertiary industries. In contrast, many elderly people (both male and female) were employed in the primary industry, as reflected in the large proportion of employed elderly individuals in the community before the earthquake.

Second, this study aimed to investigate the association between employment status and mental health conditions considering the structural industry characteristics of the affected coastal area. There was no significant difference in psychological distress measured by the K-6 in the three subgroups regarding employment status. Analysis of covariance using age as a confounding factor also indicated that there was no significant difference in posttraumatic stress reactions measured by the IES-R in the three subgroups. These results did not endorse previous findings that unemployment was associated with posttraumatic stress symptoms or poor mental health outcomes.

In contrast, this study indicated that symptoms of sleep disturbance scored by the AIS were significantly different in the subgroups regarding employment status. One-way ANOVA, followed by Tukey–Kramer’s HSD tests, indicated that the level of insomnia of the subgroup that became unemployed after the disaster (AIS score = 6.11) was significantly higher than that of the subgroup that continued to work after the disaster (AIS score = 4.57), as well as that of the subgroup that had not worked before the disaster (AIS score = 4.76). As elderly subjects were significantly dominant in the subgroup that became unemployed, and elderly people are generally likely to have insomnia [25,26], analysis of covariance was applied to the data using age as a confounding factor, showing that there remained a significant difference in AIS scores among the three groups. An interaction was not observed between age and employment status, suggesting that employment status and age were independently associated with insomnia. Changes in employment status were due to earthquake-affected sleep states, although the difference in age distribution was considered. Previous studies have indicated that disasters largely altered the sleep conditions of the affected people [9,10,11]. As the analysis was based on cross-sectional data, there was a causative relationship between employment status and sleep conditions. The relationship between becoming unemployed after the disaster and poor sleep conditions could be bidirectional; i.e., becoming unemployed after the earthquake could have exacerbated sleep disturbances and insomnia, adversely affecting employment status.

Previous research has indicated significant associations between factors such as unemployment or poor socioeconomic conditions and the symptoms of posttraumatic stress disorder [12,13,14], whereas our study did not show a significant association between employment status and posttraumatic stress reactions. It is noteworthy that there was a significant association between employment status and posttraumatic stress reactions when the ages of subjects were not considered as a confounding factor in our study. However, in the study, significant differences both in the proportion of subjects who became unemployed after the disaster and in the level of posttraumatic stress reaction were observed between elderly and younger populations, urging us to consider age as a confounding factor strongly correlated both with employment status and with posttraumatic stress reactions. Some of the previous studies did not consider age as a confounding factor, and in other studies, details of the differences in age distributions among subgroups regarding employment status were not described. Some of the studies might have overlooked the potential confounding effect of age on the association between employment status and posttraumatic stress reactions. Another possibility is that the failure to replicate the previous significant association between employment status and posttraumatic stress reactions was due to the characteristics of the communities in the current study, in which a relatively larger proportion of aged people used to work in the primary industry, which was largely affected by the disaster.

After disasters, the impact of the event on employment and economic conditions might need to be evaluated, considering the regional characteristics of the affected communities. This study suggests that, when disasters occur in coastal and mountainous areas where elderly men and women tend to be engaged in primary industries, elderly people, especially elderly women, might inevitably lose their jobs. In designing support systems for recovery after a disaster, the characteristics of the pre-disaster industrial structure of the affected communities must be considered. Employment support might be needed for subpopulations of affected communities that have vulnerable employment status. For the majority of seaside communities in the Tohoku region devastated by the Great East Japan Earthquake, elderly people who were engaged in the primary industries had a vulnerable employment status. Especially after a disaster, employment is important for maintaining good mental health conditions, not only from the aspect of earning income to reconstruct the foundations of life but also concerning increasing opportunities to interact with others. It is desirable to integrate support for employment and for mental health conditions, including guidance for maintaining good sleep. Integrative care for employment and sleep conditions might be needed for the elderly, especially elderly women in the area affected by the disaster.

## 5. Limitations and Future Lines of Research

There were several limitations to this study. First, the primary purpose of this study was to understand the changes in employment status before and after the Great East Japan Earthquake by gender, age, and type of industry. While this study included preliminary analyses of the relationship between changes in employment status and mental health conditions, the considered factors were limited. Factors including experiences of disasters and physical-health conditions [27,28], the degree of damage to houses [29], connections with people [30], history of mental illness and trauma, and the presence or absence of living with the family were not considered in this study. It is necessary to consider these remaining factors before drawing conclusions about the relationship between employment status and mental health conditions.

Second, although we examined employment status before and after the earthquake, we did not investigate the reasons for these changes. It is uncertain whether the reason for becoming unemployed was the direct impact of the earthquake. Third, this study was a cross-sectional analysis of the results of a survey conducted in 2011. It is necessary to conduct longitudinal analyses of the results of surveys conducted in consecutive years to estimate the causal relationship between employment status and mental health conditions.

## 6. Conclusions

This study pointed out the severe impact of the Great East Japan Earthquake on the employment and economic status of populations who had belonged to the primary industry, the elderly, and women. Becoming unemployed after the disaster was significantly associated with sleep disturbances.

## Figures and Tables

**Table 1:** Employment status of subjects before the Great East Japan Earthquake.

Gender	Age	The Number (%) of Subjects with Each Employment Status before the Disaster				Total	
		Unemployed		Employed			
Men	<65	75	15.6%	406	84.4%	481	100.0%
	≥65	131	57.2%	98	42.8%	229	100.0%
	Total of both age groups	206	29.0%	504	71.0%	710	100.0%
Women	<65	159	32.6%	328	67.4%	487	100.0%
	≥65	225	75.8%	72	24.2%	297	100.0%
	Total of both age groups	384	49.0%	400	51.0%	784	100.0%
Total of all subjects		590	39.5%	904	60.5%	1494	100.0%

The number of people (percentage of total subjects, *n* = 1494) who were employed/unemployed before the Great East Japan Earthquake and their subgroups (men younger than 65 years old, men equal to or older than 65 years old, women younger than 65 years old, women equal to or older than 65 years old).

**Table 2:** Employment status of subjects after the Great East Japan Earthquake.

Gender	Age	The Number (%) of Subjects with Each Employment Status after the Disaster among Those Who Were Employed before the Disaster								Total	
		Subjects Who Continued to Work		Subjects Who Obtained a New Job		Subjects Who Became Unemployed		Subject without Information of Employment Status			
Men	<65	337	83.0%	24	5.9%	37	9.1%	8	2.0%	406	100.0%
	≥65	58	59.2%	2	2.0%	33	33.7%	5	5.1%	98	100.0%
	Total of both age groups	395	78.4%	26	5.2%	70	13.9%	13	2.6%	504	100.0%
Women	<65	227	69.2%	22	6.7%	68	20.7%	11	3.4%	328	100.0%
	≥65	27	37.5%	0	0.0%	35	48.6%	10	13.9%	72	100.0%
	Total of both age groups	254	63.5%	22	5.5%	103	25.8%	21	5.3%	400	100.0%
Total of all subjects		649	71.8%	48	5.3%	173	19.1%	34	3.8%	904	100.0%

The number of subgroups (percentage of total subjects who worked before the Great East Japan Earthquake, *n* = 904) regarding employment status after the disaster—subjects: (1) who continued to work; (2) who obtained a new job; (3) who became unemployed; and (4) without information about employment status after the disaster and their subgroups (men younger than 65 years old, men equal to or older than 65 years old, women younger than 65 years old, women equal to or older than 65 years old).

**Table 3:** The number (%) of subjects with each type of industry among those who were employed before the Great East Japan Earthquake.

Gender	Age	The Number (%) of Subjects with Each Type of Industry among Those Who Were Employed before the Disaster								Total	
		The Primary Industry		The Secondary Industry		The Tertiary Industry		Miscellaneous			
Men	<65	48	11.5%	126	30.1%	218	52.0%	27	6.4%	419	100.0%
	≥65	64	57.1%	16	14.3%	29	25.9%	3	2.7%	112	100.0%
	Total of both age groups	112	21.1%	142	26.7%	247	46.5%	30	5.6%	531	100.0%
Women	<65	33	9.3%	59	16.7%	215	60.7%	47	13.3%	354	100.0%
	≥65	37	49.3%	3	4.0%	29	38.7%	6	8.0%	75	100.0%
	Total of both age groups	70	16.3%	62	14.5%	244	56.9%	53	12.4%	429	100.0%
Total of all subjects		182	19.0%	204	21.3%	491	51.1%	83	8.6%	960	100.0%

The number of subjects with each type of industry among those who were employed before the Great East Japan Earthquake (percentage of the number of subjects who were employed in each type of industry among total subjects) and their subgroups (men younger than 65 years old, men equal to or older than 65 years old, women younger than 65 years old, women equal to or older than 65 years old). When one subject was engaged in multiple jobs, the subject was redundantly counted in the respective types of industries.

**Table 4:** The number (%) of subjects who became unemployed after the Great East Japan Earthquake in subjects who were employed in each type of industry before the disaster.

Type of Industry	Gender	Age	The Number of Subjects Who Were Employed before the Disaster	The Number (%) of Subjects Who Became Unemployed after the Disaster	
The primary industry	Men	<65	48	11	22.9%
		≥65	64	25	39.1%
		Total of both age groups	112	36	32.1%
	Women	<65	33	17	51.5%
		≥65	37	16	43.2%
		Total of both age groups	70	33	47.1%
	Total of both genders		182	69	37.9%
The secondary industry	Men	<65	126	7	5.6%
		≥65	16	7	43.8%
		Total of both age groups	142	14	9.9%
	Women	<65	59	16	27.1%
		≥65	3	1	33.3%
		Total of both age groups	62	17	27.4%
	Total of both genders		204	31	15.2%
The tertiary industry	Men	<65	218	22	10.1%
		≥65	29	7	24.1%
		Total of both age groups	247	29	11.7%
	Women	<65	215	34	15.8%
		≥65	29	16	55.2%
		Total of both age groups	244	50	20.5%
	Total of both genders		491	79	16.1%
Miscellaneous	Men	<65	27	1	3.7%
		≥65	3	0	0.0%
		Total of both age groups	30	1	3.3%
	Women	<65	47	4	8.5%
		≥65	6	2	33.3%
		Total of both age groups	53	6	11.3%
	Total of both genders		83	7	8.4%
Total of all subjects			960	186	19.4%

Number (percentage) of subjects who became unemployed after the Great East Japan Earthquake among subjects who were employed in each type of industry before the disaster and their subgroups (men younger than 65 years old, men equal to or older than 65 years old, women younger than 65 years old, women equal to or older than 65 years old). When one subject was engaged in multiple jobs, the subject was redundantly counted in the respective types of industries.

**Table 5:** Changes in income among subjects who continued to work at the same workplace after the Great East Japan Earthquake.

Type of Industry	Gender	Age	The Number (%) of Subjects Who Reported Increased Income		The Number (%) of Subjects Who Reported Decreased Income		The Number (%) of Subjects Who Reported No Change in Income		Total Number (%) of Subjects Who Continued to Work at the Same Workplace after the Disaster	
The primary industry	Men	<65	2	6.9%	18	62.1%	9	31.0%	29	100.0%
		≥65	1	3.1%	14	43.8%	17	53.1%	32	100.0%
		Total of both age groups	3	4.9%	32	52.5%	26	42.6%	61	100.0%
	Women	<65	0	0.0%	7	70.0%	3	30.0%	10	100.0%
		≥65	0	0.0%	7	50.0%	7	50.0%	14	100.0%
		Total of both age groups	0	0.0%	14	58.3%	10	41.7%	24	100.0%
	Total of both genders		3	3.5%	46	54.1%	36	42.4%	85	100.0%
The secondary industry	Men	<65	21	18.4%	17	14.9%	76	66.7%	114	100.0%
		≥65	0	0.0%	3	33.3%	6	66.7%	9	100.0%
		Total of both age groups	21	17.1%	20	16.3%	82	66.7%	123	100.0%
	Women	<65	1	2.6%	10	26.3%	27	71.1%	38	100.0%
		≥65	0	0.0%	1	50.0%	1	50.0%	2	100.0%
		Total of both age groups	1	2.5%	11	27.5%	28	70.0%	40	100.0%
	Total of both genders		22	13.5%	31	19.0%	110	67.5%	163	100.0%
The tertiary industry	Men	<65	10	5.4%	47	25.5%	127	69.0%	184	100.0%
		≥65	0	0.0%	6	27.3%	16	72.7%	22	100.0%
		Total of both age groups	10	4.9%	53	25.7%	143	69.4%	206	100.0%
	Women	<65	2	1.2%	34	20.9%	127	77.9%	163	100.0%
		≥65	0	0.0%	7	58.3%	5	41.7%	12	100.0%
		Total of both age groups	2	1.1%	41	23.4%	132	75.4%	175	100.0%
	Total of both genders		12	3.1%	94	24.7%	275	72.2%	381	100.0%
Miscellaneous	Men	<65	0	0.0%	9	39.1%	14	60.9%	23	100.0%
		≥65	0	0.0%	0	0.0%	3	100.0%	3	100.0%
		Total of both age groups	0	0.0%	9	34.6%	17	65.4%	26	100.0%
	Women	<65	0	0.0%	4	14.8%	23	85.2%	27	100.0%
		≥65	0	0.0%	1	100.0%	0	0.0%	1	100.0%
		Total of both age groups	0	0.0%	5	17.9%	23	82.1%	28	100.0%
	Total of both genders		0	0.0%	14	25.9%	40	74.1%	54	100.0%
Total of all subjects			37	5.4%	185	27.1%	461	67.5%	683	100.0%

Changes in income among subjects who continued to work at the same workplace after the Great East Japan Earthquake and their subgroups regarding the type of industry before the disaster, as well as subgroups (men younger than 65 years old, men equal to or older than 65 years old, women younger than 65 years old, women equal to or older than 65 years old). When one subject was engaged in multiple jobs, he or she was counted redundantly in the respective types of industries.

**Table 6:** The differences in symptoms of each mental health condition between subgroups of residents affected by the Great East Japan Earthquake regarding employment status before and after the disaster.

	The Group that Had Not Worked before the Disaster			The Group that Became Unemployed after the Disaster			The Group that Continued to Work after the Disaster			ANOVA		HSD	ANCOVA	
	*n*	Mean	SD	*n*	Mean	SD	*n*	Mean	SD	F	*p* (Prob > F)		F	*p* (Prob > F)
AIS	299	4.76	4.05	160	6.11	3.99	629	4.57	3.50	10.88	<0.01 *	a, b	8.60	<0.01 *
K-6	301	5.26	4.84	158	5.90	4.86	628	4.97	4.56	2.51	0.08			
IES-R	296	21.07	16.61	158	22.22	15.57	622	17.94	14.77	7.17	<0.01 *	a, b	1.53	0.22

The differences in symptoms of each mental health condition, measured using the Athens insomnia scale (AIS), the Kessler 6 (K-6) psychological distress scale, and the impact-of-event scale-revised (IES-R), among the three groups, (1) that had not worked before the disaster; (2) that became unemployed after the disaster; and (3) that continued to work after the disaster, were evaluated using the analysis of variance (ANOVA). As there was a significant difference in the age of subjects that made up each group, the analysis of covariance (ANCOVA) was applied to the comparisons of AIS and IES-R scores among the three groups using age as a confounding factor. HSD: honestly significant difference, SD: standard deviation, (**a**): the group that had not worked before the disaster vs. the group that continued to work after the disaster (*p* < 0.01), (**b**): the group that became unemployed after the disaster vs. the group that continued to work after the disaster (*p* < 0.01). *: considered to be statistically significant.

## Statement

Detailed data are available for access upon request. In these cases, some of the information is excluded from the raw data to protect personal information.
